# Circulating miR-21 as a prognostic biomarker in HCC treated by CT-guided high-dose rate brachytherapy

**DOI:** 10.1186/s13014-023-02316-2

**Published:** 2023-07-28

**Authors:** Matthias Stechele, Henrike Link, Heidrun Hirner-Eppeneder, Marianna Alunni-Fabbroni, Moritz Wildgruber, Lukas Salvermoser, Stefanie Corradini, Regina Schinner, Najib Ben Khaled, Daniel Rössler, Eithan Galun, Shraga Nahum Goldberg, Jens Ricke, Philipp Maximilian Kazmierczak

**Affiliations:** 1 Department of Radiology, University Hospital, LMU Munich, Marchioninistr. 15, 81377 Munich, Germany; 2 Department of Radiation Oncology, University Hospital, LMU Munich, Munich, Germany; 3 Department of Medicine II, University Hospital, LMU Munich, Munich, Germany; 4 Goldyne Savad Institute of Gene Therapy and Division of Image-Guided Therapy and Interventional Oncology, Department of Radiology, Hadassah Hebrew University Hospital, Jerusalem, Israel

**Keywords:** microRNA, HCC, Interventional oncology, Biomarker

## Abstract

**Background and aims:**

Prognostic biomarkers identifying patients with early tumor progression after local ablative therapy remain an unmet clinical need. The aim of this study was to investigate circulating miR-21 and miR-210 levels as prognostic biomarkers of HCC treated by CT-guided high-dose rate brachytherapy (HDR-BT).

**Materials and Methods:**

24 consecutive HCC patients (BCLC A and B) treated with CT-guided HDR-BT (1 × 15 Gy) were included in this prospective IRB-approved study. RT-PCR was performed to quantify miR-21 and miR-210 levels in blood samples acquired prior to and 2 d after HDR-BT. Follow-up imaging (contrast-enhanced liver MRI and whole-body CT) was performed in 3 months follow-up intervals. Therapy response was assessed with patients classified as either responders or non-responders (12 each). Responders were defined as having no local or diffuse systemic progression within 6 months and no diffuse systemic progression exceeding 3 nodules/nodule diameter > 3 cm from 6 months to 2 years. Non-responders had recurrence within 6 months and/or tumor progression with > 3 nodules or individual lesion diameter > 3 cm or extrahepatic disease within two years, respectively. Biostatistics included parametric and non-parametric testing (Mann–Whitney-U-test), as well as Kaplan–Meier curve construction.

**Results:**

The responder group demonstrated significantly decreasing miR-21 values 2 d post therapy compared to non-responders (median miR-21 2^−ΔΔCт^: responders 0.73 [IQR 0.34], non-responders 1.53 [IQR 1.48]; *p* = 0.0102). miR-210 did not show any significant difference between responders and non-responders (median miR-210 2^−ΔΔCт^: responders 0.74 [IQR 0.45], non-responders 0.99 [IQR 1.13]; *p* = 0.8399). Kaplan–Meier curves demonstrated significantly shorter time to systemic progression for increased miR-21 (*p* = 0.0095) but not miR-210 (*p* = 0.7412), with events accumulating > 1 year post therapy in non-responders (median time to systemic progression 397 days).

**Conclusion:**

Increasing circulating miR-21 levels are associated with poor response and shorter time to systemic progression in HDR-BT-treated HCC. This proof-of-concept study provides a basis for further investigation of miR-21 as a prognostic biomarker and potential stratifier in future clinical trials of interventional oncology therapies.

*Trial registration:* In this monocentric clinical study, we analyzed prospectively acquired data of 24 patients from the “ESTIMATE” patient cohort (Studiennummer: DRKS00010587, Deutsches Register Klinischer Studien). Ethical approval was provided by the ethics committee “Ethikkommission bei der LMU München” (reference number “17-346”) on June 20, 2017 and August 26, 2020.

## Background

Image-guided local tumor ablation is a mainstay in the modern interdisciplinary, multimodal treatment of hepatocellular carcinoma (HCC). According to the current European Society of Medical Oncology (ESMO) guidelines, local tumor ablation using radiofrequeny ablation (RFA) is a first-line treatment option in very early-stage [1] (Barcelona Clinic Liver Cancer/BCLC 0) and early-stage HCC (BCLC A, up to three nodules ≤ 3 cm). As an alternative to RFA, computed tomography (CT)-guided high-dose rate brachytherapy (HDR-BT) may be performed, with excellent local tumor control rates and safety profile [1–3]. Compared to RFA, HDR-BT is not limited by tumor size, exophytic tumor growth, or the heat-sink effect [1]. Despite favorable hepatic tumor control and survival rates achieved by local tumor ablation, response prediction, i.e. early differentiation of responders and non-responders, remains an unmet clinical need. Furthermore, prognostic biomarkers identifying patients with early local or systemic tumor progression after local ablative therapy would potentially allow for an individualized administration of adjuvant drugs to optimize therapy response.

In recent years, micro-ribonucleic acids (miRs) have gained attention as regulators of complex biological functions and processes in various mammalian cells [4]. miRs are short non-coding nucleic acids with 18–24 nucleotides found within cells and circulating in the blood [5]. miR-21 is a key player in various types of liver disease, including alcoholic liver disease, non-alcoholic fatty liver disease, fibrosis and HCC [6, 7]. It was demonstrated that high miR-21 expression is associated with tumor progression in HCC, which is predominantly mediated by phosphatase and tensin homolog (PTEN) [8–11]. Induced by hypoxia, miR-210 is another miR involved in HCC tumor progression [12, 13]. miR-210 has been shown to induce tumor angiogenesis in HCC by activation of fibroblast growth factor receptor-like 1 (FGFRL1) [13]. Both miR-21 and miR-210 demonstrate increased plasma levels shortly after thermal ablation of HCC nodules, peaking at 60–90 min post intervention and normalizing within one week [14]. However, it remains to be determined whether plasma levels of miR-21 and miR-210 yield prognostic value with regard to patient outcome in HDR-BT-treated HCC. Accordingly, the aim of this prospective study was to investigate the potential of plasma miR-21 and miR-210 as prognostic biomarkers in CT-guided HDR-BT. We hypothesized that plasma levels of miR-21 and miR-210 before and 48 h after local ablation allow for response prediction in HCC patients treated with CT-guided HDR-BT.

## Materials and methods

### Study design and eligibility criteria

This is an analysis of patients from a prospective cohort investigating the systemic effects of HDR-BT in HCC. Patients were recruited between August 2017 and November 2019 and provided written informed consent for both the local ablative treatment and study inclusion. Median follow-up was 15 months (6–40 months/183–1224 days, mean 20 months). Eligibility criteria included previously untreated HCC stage BCLC A and B and absence of any immunodeficiency or immunosuppressive therapy such as cortisone treatment up to two weeks prior to study inclusion. With regard to tumor size, HDR-BT allowed for inclusion of larger tumors up to 10 cm.

### Study procedures

Prior to local tumor ablation, contrast-enhanced CT of chest, abdomen, and pelvis as well as Gd-EOB-DTPA-enhanced liver MRI (Primovist^®^, Bayer Vital GmbH Gb Pharma, Leverkusen, Germany) was performed for tumor detection and staging. Before HDR-BT, local anesthesia (lidocaine) as well as intravenous analgesia (fentanyl) and sedation (midazolam) were administered at weight-adapted doses and with regard to individual discomfort and pain levels. Subsequently, the target tumors were punctured using an 18-gauge needle (Bard^®^ Mission™, Disposable Core Biopsy Instrument, Bard Peripheral Vascular Inc, Tempe, AZ, 18 Gauge, 2 cores each, penetration depth 10 and 20 mm) under CT fluoroscopic guidance (SOMATOM Edge, Siemens Healthineers AG, Forchheim, Germany). A flexible 6-French catheter sheath (Radifocus, Terumo, Tokyo, Japan) was introduced over a rigid angiographic guidewire (Amplatz, Boston Scientific, Marlborough, USA) using Seldinger technique. Then, a 6-French afterloading catheter (Primed Medizintechnik Gmbh, Halberstadt, Germany) was inserted and the extracorporeal portion of the catheter was temporarily sutured to the skin. The angulation and number of catheters were determined individually according to the size of the target tumor while taking into consideration organs at risk in close proximity. Finally, a contrast-enhanced CT scan of the liver was obtained to confirm the correct catheter positioning and to plan the subsequent high-dose rate irradiation. The clinical target volume (CTV) and the adjacent organs at risk (OAR, e.g., gastrointestinal tract) were delineated by a radiation oncologist using the planning software system Oncentra (Nucletron, Elekta Ab, Stockholm, Sweden). Usually, no additional planning target volume (PTV) margins were added. For treatment planning, all cathethers were correctly identified and the three-dimensional coordinates (x, y, z) were reconstructed in the treatment planning system. Dose optimization was performed using inverse or graphical optimization with an aimed prescription dose of 15 Gy in a single fraction. Sparing of OARs with fulfilment of the corresponding dose constraints was priorized over full dose coverage. The dose was delivered using a HDR brachytherapy afterloading system (Nucletron, Elekta Ab, Stockholm, Sweden) with an iridium-192 source. After completion of the irradiation, the catheters were removed and the puncture tracts were filled with gel foam. Patients then remained in our postinterventional observation unit for 2 h before being transferred to the ward.

Peripheral blood was obtained on the day before therapy and 48 h after HDR-BT. 5 mL were collected in Monovette EDTA tubes (Sarstedt AG, Nümbrecht, Germany) and centrifuged within 1 h after blood draw (3000 rpm, 5 min, 4 °C). Plasma was immediately aliquoted and stored at − 80 °C till use.

### Quantification of miR-21 and miR-210

The MagMAX™ mirVana™ Total RNA Isolation Kit (ThermoFisher Scientific, Darmstadt, Germany) was used for RNA isolation according to the manufacturer´s instructions. Briefly, after digestion of plasma (100 µl) with Proteinase K, RNA purification was done using RNA binding beads and a magnet stand. Samples were treated with TURBO DNase™ and finally RNA was eluted in 50 µl of pre-heated Elution Buffer. A Nanodrop spectrophotometer (Implen, Munich, Germany) was used to determine the amount of RNA. RNA was stored at − 20 °C or directly reverse transcribed to cDNA utilizing the TaqMan^®^ Advanced miRNA cDNA Synthesis Kit (ThermoFisher Scientific). The following TaqMan^®^ Advanced miRNA Assays (ThermoFisher Scientific) were used for relative miR quantification: hsa-miR-21-3p (477973-mir), hsa-miR-210-3p (477970-mir); as endogenous controls, hsa-miR-16-5p (477860-mir), hsa-miR-19a-5p (479228-mir) and hsa-miR-26a-5p (477995-mir). Correlation coefficient (R^2^) and PCR efficiency calculated from slope were all between 0.977–0.990 and 92–128%, respectively. All qPCR reactions were run on 96-well plates on a Quant Studio 5 Fast Real-Time PCR System (ThermoFisher Scientific). For each assay, all samples were run in triplicate.

### Definition of responders and non-responders

Patients were stratified into responders and non-responders based on previously published criteria for HCC disease stages and eligibility for curative versus palliative treatments in cases of progression [15]. Briefly, responders were defined as having no limited (up to BCLC A) or diffuse systemic tumor progression within 6 months and no diffuse systemic progression exceeding 3 nodules/nodule diameter > 3 cm after 6 months to 2 years. Non-responders had recurrence within 6 months and/or tumor progression with > 3 nodules or individual nodule diameter > 3 cm or extrahepatic disease within two years, respectively. In two patients, liver transplantation occurred 9 months after local tumor ablation. In this follow-up period, these patients had no tumor recurrence. Furthermore, the explanted livers did not show evidence for new viable tumor in pathological examination—thus, these patients were stratified as “responders”.

### Statistical analysis

**Fig. 1:**
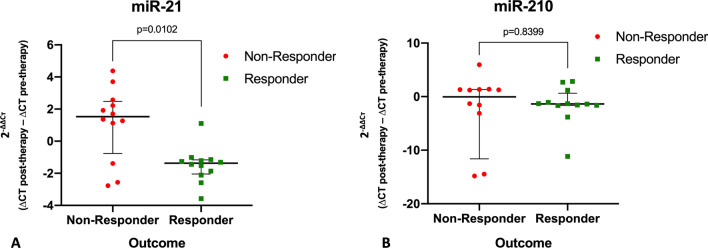
miR-21 (**a**) and miR-210 (**b**) values in responders and non-responders. To enable optimized depiction (i.e. decreasing miR values displayed as negative numbers), 2^−ΔΔCт^ values between 0 and 1 (implicating decrease) were transformed applying the formula: 1/− (2^−ΔΔCт^). Note the significant miR-21 level decrease after local ablative therapy in the responders group compared to the non-responders group. miR-210 levels did not show a significant change. Note: For miR-210, lower range of the y-axis was set to − 20 to allow for optimized comparability of responders and non-responders. Therefore, patient no. 21 (transformed 2^−ΔΔCт^ = − 34) is not displayed. MWU = Mann–Whitney U Test

**Fig. 2:**
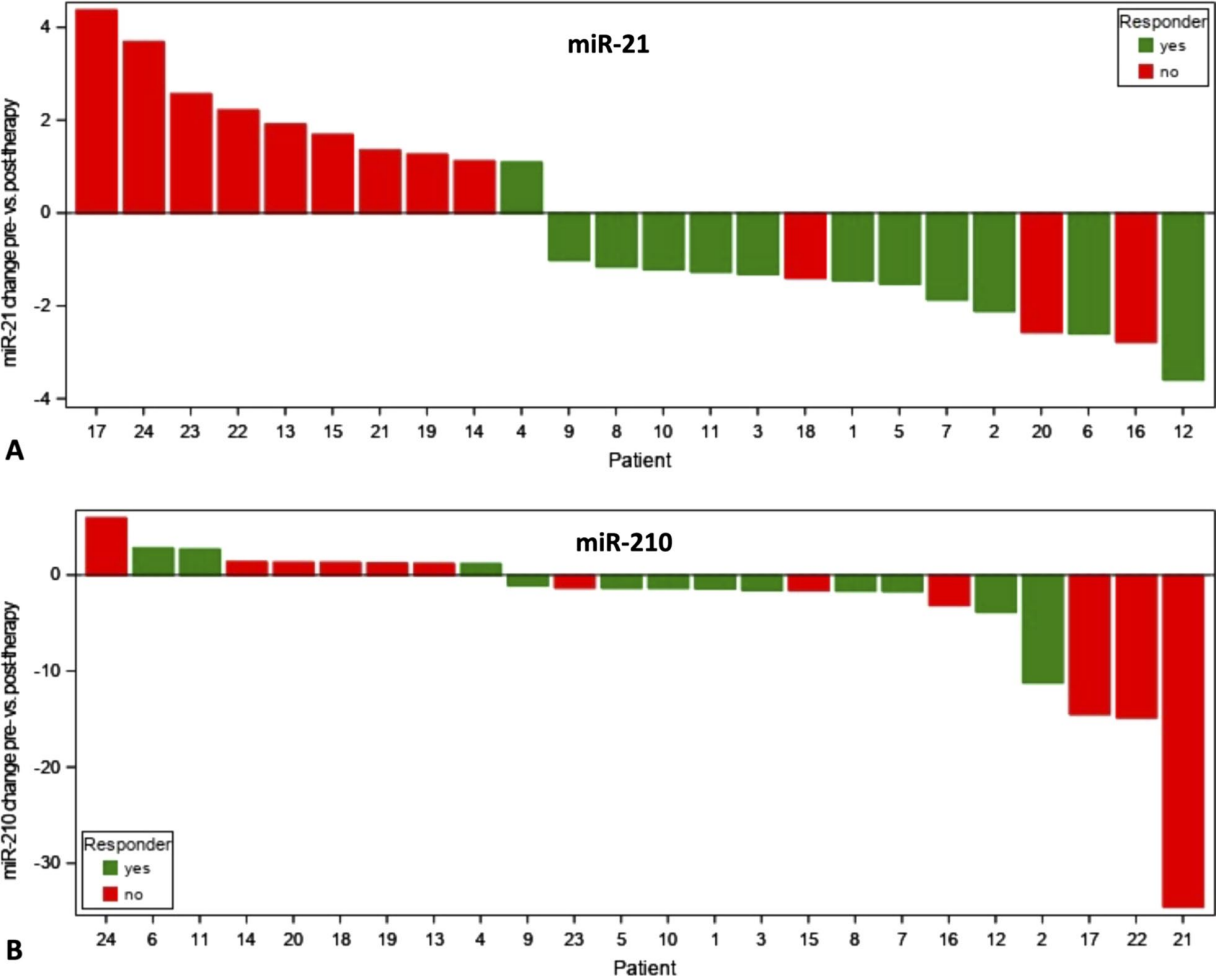
Waterfall plots illustrating miR dynamics per individual study subject for miR-21 (**a**) and miR-210 (**b**). To enable optimized depiction (i.e. decreasing miR values displayed as negative numbers), 2^−ΔΔCт^ values between 0 and 1 (implicating decrease) were transformed applying the formula: 1/− (2^−ΔΔCт^). Note the significant increase of miR-21 in non-responders

**Fig. 3:**
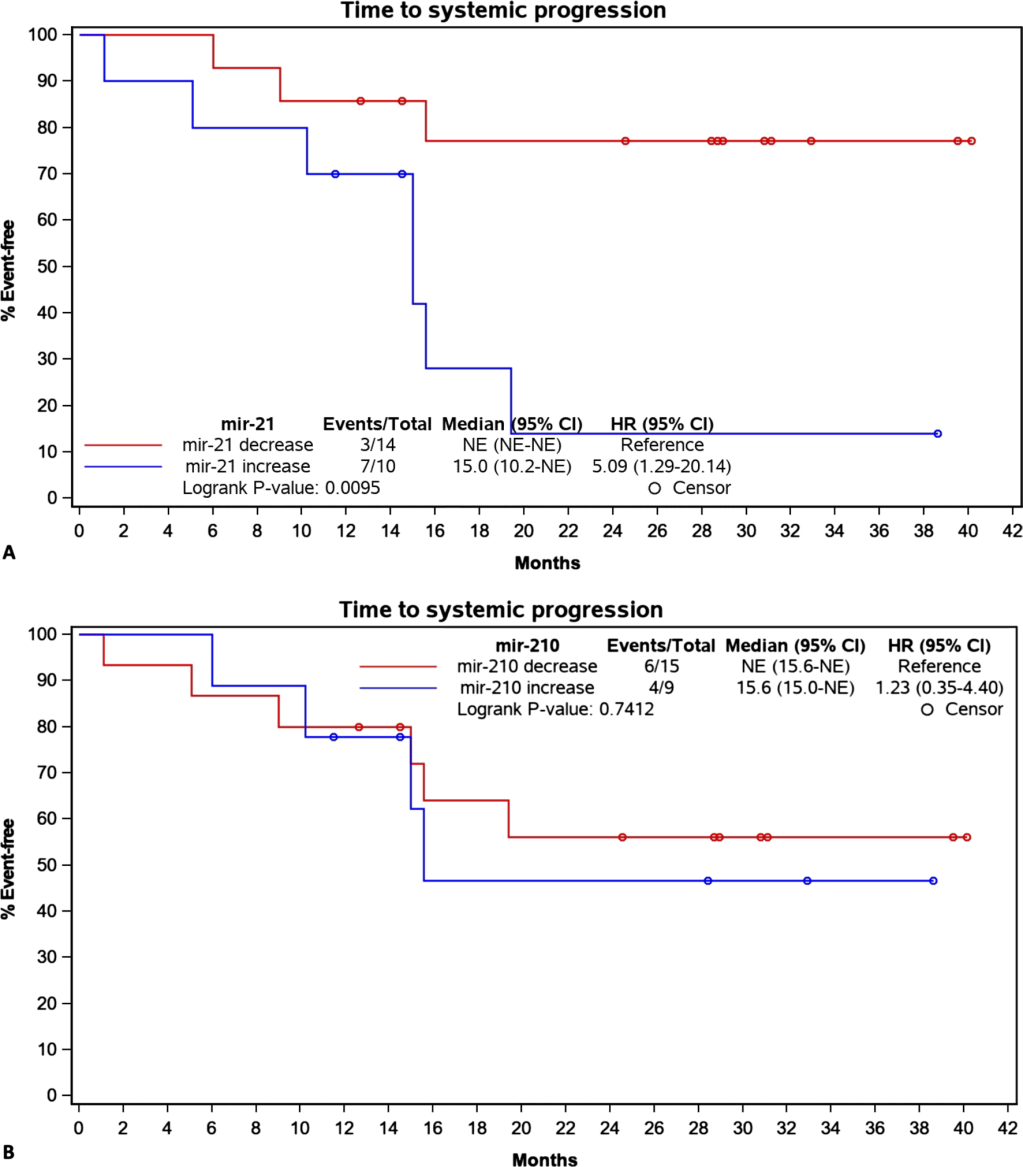
Kaplan–Meier curves demonstrating time to systemic progression in relation to increasing and decreasing miR-21/-210 levels. Note the significantly shorter time to systemic progression in patients with increasing miR-21 levels (**A**). No significant difference in time to systemic progression between increasing and decreasing miR-210 levels was observed (**B**). NE – not evaluable because median time to systemic progression was not reached within the 40 months of study follow-up

miR levels were quantified using the 2^−ΔΔCт^ method as previously described [16]. Categorical data were reported with numbers and percentages and group differences in dichotome variables were checked using Fisher’s exact test. Continuous data were checked for normal distribution using the Shapiro–Wilk test. Normally distributed data were reported as means ± standard deviations and according group differences were evaluated using a t-test. Non-normally distributed data were reported as medians (range) and according group differences were evaluated using the Mann–Whitney U test. Spearman correlation was applied for correlation of laboratory parameters and technical HDR-BT data with miR changes. Time to systemic progression was evaluated by the Kaplan–Meier method and group differences were compared using a log-rank test. In patients with no systemic progression, the data was censored at the date of last available follow-up. Statistical significance was assumed for *p* < 0.05. The statistical analysis and creation of Figs. 2 and 3 was performed using dedicated software (SAS version 9.4 for Windows, SAS Institute Inc., Cary, NC, USA). Figure 1 was created using GraphPad Prism Version 9.5.1 (GraphPad Software, Boston, MA, USA).

## Results

### Patient population

Twenty four patients were recruited. Patient characteristics are provided in Table 1. No statistically significant differences in baseline parameters were observed between responders and non-responders.

**Table 1:** Patient characteristics

Baseline features	Overall		Responders (n = 12)		Non-resp. (n = 12)		*p*-value
	Number/Median	Range (IQR) %	Number/Median	Range (IQR)/%	Number/Median	Range (IQR)/%	
*Sex*							
Female	4	16.7	2	16.7	2	16.7	1
Male	20	83.3	10	83.3	10	83.3	1
Age (years)	69	50–84 (21)	68.5	58–87 (21.5)	68.5	44–86 (32.5)	0.80
*Etiology*							
Alcohol	9	37.5	5	41.7	4	33.3	1
NASH	3	12.5	1	8.3	2	16.7	1
Hepatitis B	4	16.7	1	8.3	3	25	0.59
Hepatitis C	6	25	4	33.3	2	16.7	0.64
Unknown	6	25	3	25	3	25	1
Multiple	4	16.7	2	16.7	2	16.7	
Child–Pugh A	19	79.2	10	83.3	9	75	1
Child–Pugh B	5	20.8	2	16.7	3	25	
AFP [ng/ml]	5.25	1.6–35,754 (7)	5.25	1.6–21.5 (4.65)	5.45	2.0–35,754 (32.15)	0.77
AFP ≥ 20 ng/ml	4	16.7	1	8.3	3	25	0.59
Portal vein thrombosis	0	0	0	0	0	0	1
Sum lesion diameter [cm]	3	1.1–10.9 (3.23)	2.9	1.7–10.3 (1.08)	3.5	1.1–10.9 (4.75)	0.49
Max. tumor diameter [cm]	2.5	1.1–10.3 (1.63)	2.7	1.7–10.3 (0.8)	2.3	1.1–10.0 (5.67)	0.31
*Tumor number treated*							
1	16	66.7	10	83.3	6	50	0.19
2	8	33.3	2	16.7	6	50	

*NASH*, non alcoholic steatohepatitis; *AFP*, alpha-fetoprotein

### Clinical outcome

Applying the above-described response criteria, there were n = 12 responders and n = 12 non-responders (Table 2). Median time to systemic progression or loss to follow up was 912 d in responders and 397 d in non-responders. In responders, 4 out of 12 patients (33%) showed limited progression at a median of 718 d, whereas 8 out of 12 (67%) were lost to follow-up (at a median of 879 d). None of the responders showed systemic progression during the follow-up period. In non-responders, 5 out of 12 patients (42%) showed limited progression at a median of 182 d, whereas 5 out of 12 (42%) showed systemic progression. Two patients (16.7%) of the non-responder group had limited progression after 182 and 138 d with last contact at 351 and 442 d which was considered as time to systemic progression.

**Table 2:** Individual values for miR-21, miR-210 values, and clinical outcome

Patient	miR-21			miR-210			TTLP (d)	TTSP (d)
	ΔCт mean pre-therapy	ΔCт mean post-therapy	2^−ΔΔCт^	ΔCт mean pre-therapy	ΔCт mean post-therapy	2^−ΔΔCт^		
*Responders*								
1	6.08	6.61	0.69	5.14	5.62	0.72	581	949
2	6.55	7.63	0.48	5.18	8.66	0.09	None	1205
3	6.96	7.34	0.76	4.72	5.35	0.65	None	443
4	6.00	5.86	1.10	7.42	7.17	1.19	854	1178
5	5.66	6.26	0.66	4.87	5.26	0.76	None	940
6	7.29	8.66	0.39	7.20	5.70	2.83	1004	1004
7	6.38	7.27	0.54	5.02	5.76	0.60	None	749
8	6.45	6.64	0.87	4.65	5.34	0.62	None	1224
9	5.79	5.80	0.99	6.25	6.34	0.94	None	875
10	5.30	5.57	0.83	5.36	5.76	0.76	None	883
11	5.93	6.27	0.79	5.94	4.51	2.70	267	867
12	5.52	7.36	0.28	5.17	7.10	0.26	None	386
*Non-responders*								
13	6.53	5.59	1.92	5.34	5.06	1.21	None	457
14	6.94	6.76	1.13	4.82	4.35	1.39	182	351
15	7.03	6.26	1.70	4.62	5.26	0.64	None	457
16	6.02	7.49	0.36	5.82	7.46	0.32	93	275
17	7.38	5.24	4.38	5.22	9.08	0.07	225	475
18	4.66	5.14	0.72	5.80	5.40	1.32	None	183
19	5.94	5.59	1.27	5.62	5.29	1.26	138	442
20	6.16	7.51	0.39	5.02	4.60	1.33	None	475
21	5.76	5.31	1.36	5.37	10.48	0.03	490	592
22	7.74	6.59	2.22	3.58	7.47	0.07	None	155
23	5.92	4.56	2.57	4.20	4.57	0.77	None	34
24	7.02	5.13	3.70	7.12	4.55	5.96	None	312

*TTLP*, Time to limited progression; *TTSP*, Time to systemic progression; 2^−ΔΔCт^ = [(Δт post-therapy) − (ΔCт pre-therapy)]; *d*: days

### miR-21 and miR-210 levels before and after CT-guided HDR-BT

There were no statistically significant differences in pre-treatment miR-21 and miR-210 levels between responders and non-responders (miR-21: ΔCт median [IQR] responders 6.04 [0.77]), non-responders 6.34 [1.09], *p* = 0.39; miR-210: responders 5.17 [1.15], non-responders 5.28 [0.99], *p* = 0.34). Responders demonstrated significantly decreasing miR-21 values 2 d after local ablative treatment compared to non-responders (median miR-21 2^−ΔΔCт^: responders 0.73 [IQR 0.34], non-responders 1.53 [IQR 1.48]; *p* = 0.0102) (Fig. 1a). miR-210 levels did not show any significant difference between responders and non-responders (median miR-210 2^−ΔΔCт^: responders 0.74 [IQR 0.45], non-responders 0.99 [IQR 1.13]; *p* = 0.8399) (Fig. 1b).

Overall, 10 out of 24 patients (42%) showed a miR-21 increase, whereas 14 out of 24 (58%) demonstrated decreasing miR-21 levels. Out of the 10 patients with increasing miR-21, 9 (90%) were non-responders and 1 (10%) was a responder. 3 out of 14 patients (21%) with decreasing miR-21 levels were non-responders, whereas 11 of 14 patients (79%) were responders (Fig. 2a).

For miR-210, overall 9 out of 24 patients (38%) showed an increase and 15 out of 24 patients (63%) a decrease after therapy. Out of 9 patients with increases, 6 (67%) were non-responders and 3 out of 9 were responders (33%). Of 15 patients with decreasing miR-210 levels, 6 (40%) were non-responders and 9 (60%) were responders (Fig. 2b).

### Correlation of baseline plasma, oncological, and HDR-BT parameters with miR-21 and miR-210 levels

Correlation between baseline plasma parameters and miR levels pre- and post-therapy showed few but weak correlations. For miR-21, only liver parameters alanine transaminase (ALAT) and aspartate transaminase (ASAT) demonstrated a slight correlation with 2^−ΔΔCт^ (Spearman’s Rho = 0.53 and 0.42 (*p* = 0.01 and 0.04, respectively). With regard to HDR-BT parameters, a weak negative correlation was found between post-treatment miR-21 levels and exposed liver volume (Spearman’s Rho = − 0.42, *p* = 0.04). Of note, miR-21 levels were independent of the irradiated liver volume (Spearman’s Rho = 0.26, *p* = 0.22). For miR-210, no statistically significant correlations were observed. Detailed correlations are provided in Table 3.

**Table 3:**
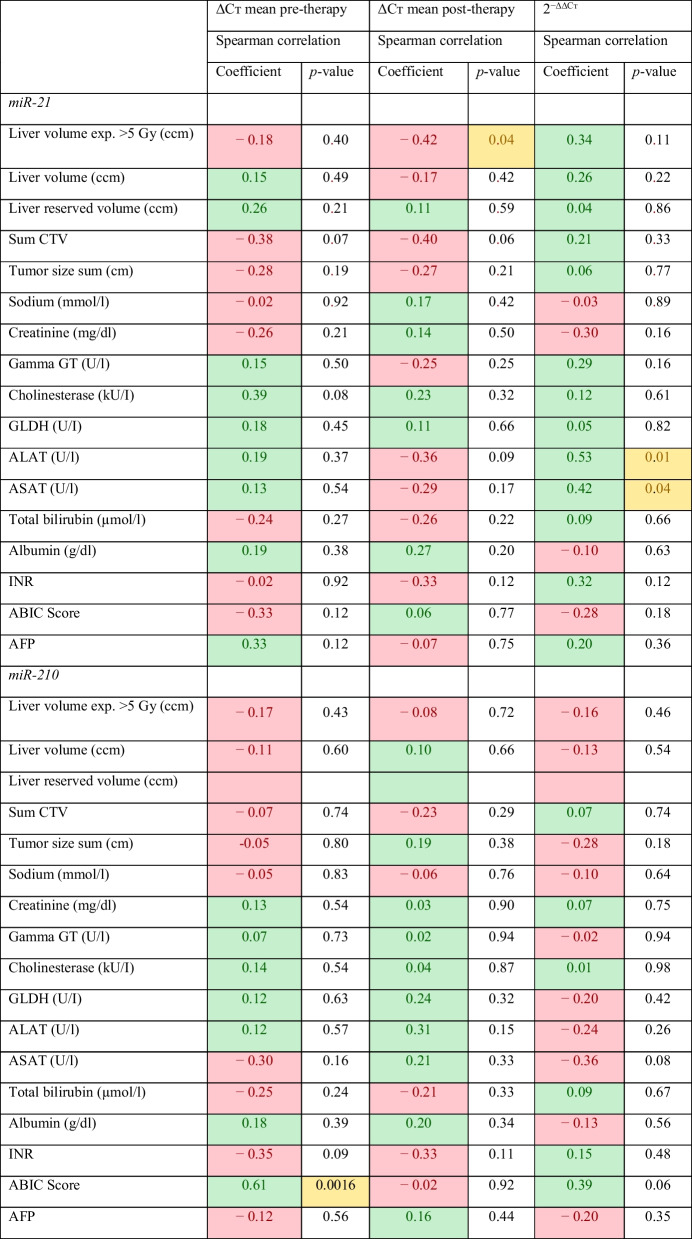
Linear correlations between miR levels and HDR-BT/clinical parameters in the investigated population

### Changes in miR-21 & miR-210 levels and oncological outcome

Median time to systemic progression was 15.0 months (95% CI 10.2–NE) in patients with miR-21 increase and was not reached in patients with miR-21 decrease within the 40 months of study follow-up (hazard ratio [HR] 5.09; 95% CI 1.29–20.14; *p* = 0.0095) (Fig. 3a). Median time to systemic progression was 15.6 months (95% CI 15.0–NE) in patients with miR-210 increase and not evaluable in patients with miR-210 decrease ([HR] 1,23; 95% CI 0.35–4.4; *p* = 0.7412) (Fig. 3b).

## Discussion

In this proof-of-concept study, we investigated circulating miR-21 and miR-210 as potential prognostic biomarkers of therapy response for HDR-BT-treated HCC. We demonstrated that increasing miR-21 plasma levels 2 d after local ablative therapy were associated with poor therapy response and shorter time to systemic progression. In contrast to miR-21, peri-interventional miR-210 plasma levels did not serve as predictors of patient outcome. The increase of miR-21 was independent of the irradiated volume of surrounding liver tissue.

Our results are in line with a recent study demonstrating significantly increased miR-21 and miR-210 plasma levels shortly after thermal ablation (RFA or microwave ablation) of HCC and colorectal carcinoma liver metastases, peaking 60–90 min after the intervention and normalizing within 7 d [14]. However, the authors showed that increases of miR-210 but not miR-21 were associated with early progressive disease after three months. Differences in the biological effect of thermal ablation and conformal radiation with regard to tumor necrosis/apoptosis as well as differences in sampling time points may be potential explanations for this discrepancy to our findings. In addition, the authors performed a pooled analysis for both HCC and colorectal carcinoma liver metastases, while only HCC patients were included in our study.

Although we found a significant association between increased circulating miR-21 and systemic tumor progression, clarification of its predictive and prognostic value in HCC will require further studies. Indeed, Franck et al. [17] did not observe a significant difference with regard to overall survival in HCC patients based upon high versus low plasma miR-21 levels. However, the authors investigated a pooled study population of 91 patients and did not differentiate between tumor burden and the therapies performed (i.e. systemic therapy, surgery, local ablation). In addition, they report a significant moderate inverse correlation of plasma miR-21 and serum creatinine and aspartate aminotransferase, pointing to kidney function and liver injury as potential influencing factors [17]. In the present study however, we did not find a significant correlation of miR-21 and the assessed laboratory parameters, excluding these as potential confounders in the investigated population.

Previous studies demonstrated that high miR-21 expression in HCC tissue is prognostic of poor survival [18, 19]. Huang et al. [18] measured miR-21 expression in 166 specimens of surgically resected HCC nodules and found high miR-21 expression as independent prognostic factor for shorter overall (HR 2.36) and disease-free survival (HR 2.02). In accordance with the aforementioned study, Zhu et al. showed that high miR-21 expression in HCC significantly correlates with short-term relapse (≤ 6 months) as well as shorter disease-free and overall survival after hepatectomy [19].

To date, evidence regarding potential outcome prediction in HCC is more compelling for intratumoral than for circulating miR-21. Although it was shown that miR-21 plasma levels parallel intratumoral miR-21 expression, potential differences between baseline miR levels in therapy-naïve patients and patients under local tumor ablation must be taken into consideration [20]. Not only did we measure baseline miR-21 and miR-210 values, but also assessed the development of individual plasma levels under therapy (i.e. before versus 48 h after the intervention). It is likely that local tumor ablation causes a release of intratumoral miR and that miR plasma levels and plasma level time course differ depending on the type of ablation and cell death (i.e. direct hyperthermic cellular damage with or without secondary apoptosis in the transitional zone after RFA versus radiation-induced cell death after HDR-BT) [21–23]. Data on the effect of ionizing radiation on miR-21 is limited. In vitro investigations revealed that irradiation increases miR-21 expression in human umbilical vein endothelial cells (HUVEC) with consecutive higher proliferation [24]. Therefore, the type of local therapy potentially influences the capability of miR as prognostic biomarkers in individual patients, which needs to be investigated in further clinical and preclinical studies.

Our study also provides a potential, initial basis for future investigation of adjuvant therapies modulating miR-21 activity and its downstream pathways after local tumor ablation. Preclinical in vitro and in vivo data show that curcumin suppresses HCC tumor growth/cell proliferation and induces apoptosis in a dose-dependent manner, partly mediated by downregulation of miR-21 expression [25]. The effect of curcumin on cell proliferation and apopotosis was increased by cellular transfection with a specific miR-21 inhibitor [25]. Similar effects of therapeutic miR-21 inhibition were reported in cervical carcinoma cells in vitro as well as in vivo in a murine melanoma and glioma model [26]. The investigation of adjuvant miR-21 inhibition to enhance therapy effects of local ablative tumor therapies remains subject to future preclinical and clinical studies.

### Limitations

We acknowledge several limitations to our study. First, it remains to be elucidated what exactly causes changes in plasma miR-21 and miR-210 after HDR-BT. Our data indicate that the post-therapeutic miR-21 increase does not originate from radiation-induced damage of the surrounding liver tissue. However, the exact mechanism including the interaction of miR-21 with its respective downstream targets needs to be further investigated. Second, we only measured levels of circulating miR but did not assess miR expression in tumor and liver tissue samples before and after HDR-BT. Third, the optimal timepoint for measurement of circulating miR-21 remains unknown. Andrasina et al. have demonstrated that miR-21 and miR-210 peak at 60–90 min after thermal ablation and at 24 h after TACE of HCC nodules, indicating that miR plasma kinetics depend on the type of local tumor ablation. Additional miR-21 quantifications at multiple timepoints after HDR-BT are needed to identify the peak of miR-21 increase following high-dose irradiation. Finally, detailed knowledge on miR-21 plasma kinetics will support us to choose the optimal timepoint for administering potential adjuvant therapeutics modulating systemic effects of local tumor ablation.

## Conclusion

In this proof-of-concept study, we demonstrated that increasing miR-21 plasma levels 2 d after HDR-BT in HCC were associated with poor therapy response and shorter time to systemic progression. Our data provide an initial basis for further investigation of miR-21 as prognostic biomarker and potential target for therapeutic modulation in local tumor ablation. However, the exact role of miR-21 in HCC and its effect on tumor progression awaits clarification in additional preclinical and clinical studies.

## Data Availability

Research data are stored in an institutional repository and will be shared upon request to the corresponding author.

## References

[1] Vogel A, Cervantes A, Chau I, Daniele B, Llovet JM, Meyer T (2019). Hepatocellular carcinoma: ESMO clinical practice guidelines for diagnosis, treatment and follow-up. Ann Oncol.

[2] Mohnike K, Wieners G, Schwartz F, Seidensticker M, Pech M, Ruehl R (2010). Computed tomography-guided high-dose-rate brachytherapy in hepatocellular carcinoma: safety, efficacy, and effect on survival. Int J Radiat Oncol Biol Phys.

[3] Collettini F, Schreiber N, Schnapauff D, Denecke T, Wust P, Schott E (2015). CT-guided high-dose-rate brachytherapy of unresectable hepatocellular carcinoma. Strahlenther Onkol.

[4] Gebert LFR, MacRae IJ (2019). Regulation of microRNA function in animals. Nat Rev Mol Cell Biol.

[5] Zhang T, Yang Z, Kusumanchi P, Han S, Liangpunsakul S (2020). Critical role of microRNA-21 in the pathogenesis of liver diseases. Front Med (Lausanne).

[6] Blaya D, Coll M, Rodrigo-Torres D, Vila-Casadesus M, Altamirano J, Llopis M (2016). Integrative microRNA profiling in alcoholic hepatitis reveals a role for microRNA-182 in liver injury and inflammation. Gut.

[7] Kitano M, Bloomston PM (2016). Hepatic stellate cells and microRNAs in pathogenesis of liver fibrosis. J Clin Med.

[8] Yoon JS, Kim G, Lee YR, Park SY, Tak WY, Kweon YO (2018). Clinical significance of microRNA-21 expression in disease progression of patients with hepatocellular carcinoma. Biomark Med.

[9] Huang CS, Yu W, Cui H, Wang YJ, Zhang L, Han F (2015). Increased expression of miR-21 predicts poor prognosis in patients with hepatocellular carcinoma. Int J Clin Exp Pathol.

[10] Meng F, Henson R, Wehbe-Janek H, Ghoshal K, Jacob ST, Patel T (2007). MicroRNA-21 regulates expression of the PTEN tumor suppressor gene in human hepatocellular cancer. Gastroenterology.

[11] Zhou Y, Ren H, Dai B, Li J, Shang L, Huang J (2018). Hepatocellular carcinoma-derived exosomal miRNA-21 contributes to tumor progression by converting hepatocyte stellate cells to cancer-associated fibroblasts. J Exp Clin Cancer Res.

[12] Ying Q, Liang L, Guo W, Zha R, Tian Q, Huang S (2011). Hypoxia-inducible microRNA-210 augments the metastatic potential of tumor cells by targeting vacuole membrane protein 1 in hepatocellular carcinoma. Hepatology.

[13] Yang Y, Zhang J, Xia T, Li G, Tian T, Wang M (2016). MicroRNA-210 promotes cancer angiogenesis by targeting fibroblast growth factor receptor-like 1 in hepatocellular carcinoma. Oncol Rep.

[14] Andrasina T, Juracek J, Zavadil J, Cechova B, Rohan T, Vesela P (2021). Thermal ablation and transarterial chemoembolization are characterized by changing dynamics of circulating MicroRNAs. J Vasc Interv Radiol.

[15] Reig M, Forner A, Rimola J, Ferrer-Fabrega J, Burrel M, Garcia-Criado A (2022). BCLC strategy for prognosis prediction and treatment recommendation: the 2022 update. J Hepatol.

[16] Livak KJ, Schmittgen TD (2001). Analysis of relative gene expression data using real-time quantitative PCR and the 2(-Delta Delta C(T)) Method. Methods.

[17] Franck M, Thon C, Schutte K, Malfertheiner P, Link A (2020). Circulating miR-21-5p level has limited prognostic value in patients with hepatocellular carcinoma and is influenced by renal function. World J Hepatol.

[18] Huang X, Xiong Y, Yang J, Yang G, Li J (2020). The prognostic significance of miR-21 expression among surgically resected hepatocellular carcinoma patients: evidence from a meta-analysis and retrospective cohort study. Biomed Res Int.

[19] Zhu C, Zhang M, Hu J, Li H, Liu S, Li T (2018). Prognostic effect of IL-6/JAK2/STAT3 signal-induced microRNA-21-5p expression on short term recurrence of hepatocellular carcinoma after hepatectomy. Int J Clin Exp Pathol.

[20] Guo X, Lv X, Lv X, Ma Y, Chen L, Chen Y (2017). Circulating miR-21 serves as a serum biomarker for hepatocellular carcinoma and correlated with distant metastasis. Oncotarget.

[21] Chu KF, Dupuy DE (2014). Thermal ablation of tumours: biological mechanisms and advances in therapy. Nat Rev Cancer.

[22] Damm R, Pech M, Haag F, Cavalli P, Gylstorff S, Omari J (2022). TNF-alpha indicates radiation-induced liver injury after interstitial high dose-rate brachytherapy. In Vivo.

[23] Damm R, Pech M, Cavalli P, Haag F, Gylstorff S, Omari J (2022). Correlation of chemokines and growth factors with radiation-induced liver injury after interstitial high dose rate (HDR) brachytherapy of liver metastases. J Cancer Res Clin Oncol.

[24] Zhang Y, Chen Z, Feng L, Jiang P, Li X, Wang X (2019). Ionizing radiation-inducible microRNA-21 induces angiogenesis by directly targeting PTEN. Asian Pac J Cancer Prev.

[25] Li J, Wei H, Liu Y, Li Q, Guo H, Guo Y (2020). Curcumin inhibits hepatocellular carcinoma via regulating miR-21/TIMP3 Axis. Evid Based Complement Alternat Med.

[26] Javanmard SH, Vaseghi G, Ghasemi A, Rafiee L, Ferns GA, Esfahani HN (2020). Therapeutic inhibition of microRNA-21 (miR-21) using locked-nucleic acid (LNA)-anti-miR and its effects on the biological behaviors of melanoma cancer cells in preclinical studies. Cancer Cell Int.

